# Indirect effect inference and application to GAW20 data

**DOI:** 10.1186/s12863-018-0638-3

**Published:** 2018-09-17

**Authors:** Liming Li, Chan Wang, Tianyuan Lu, Shili Lin, Yue-Qing Hu

**Affiliations:** 1 State Key Laboratory of Genetic Engineering, Institute of Biostatistics, School of Life Sciences, Fudan University, 2005 Songhu Road, Shanghai, 200438 China; 2 Department of Statistics, The Ohio State University, 1958 Neil Avenue, 404 Cokins Hall, Columbus, OH 43210 USA

**Keywords:** Epigenetics, Differentially methylated regions, DNA methylation

## Abstract

**Background:**

Association studies using a single type of omics data have been successful in identifying disease-associated genetic markers, but the underlying mechanisms are unaddressed. To provide a possible explanation of how these genetic factors affect the disease phenotype, integration of multiple omics data is needed.

**Results:**

We propose a novel method, LIPID (likelihood inference proposal for indirect estimation), that uses both single nucleotide polymorphism (SNP) and DNA methylation data jointly to analyze the association between a trait and SNPs. The total effect of SNPs is decomposed into direct and indirect effects, where the indirect effects are the focus of our investigation. Simulation studies show that LIPID performs better in various scenarios than existing methods. Application to the GAW20 data also leads to encouraging results, as the genes identified appear to be biologically relevant to the phenotype studied.

**Conclusions:**

The proposed LIPID method is shown to be meritorious in extensive simulations and in real-data analyses.

## Background

In complex disease studies, genome-wide association studies (GWAS) [1] and epigenome-wide association studies [2] have been successful in identifying disease-associated single-nucleotide polymorphisms (SNPs) and DNA methylation loci. However, the mechanism of how these genetic loci affect the disease status remains unknown. To provide a possible explanation of the causal mechanisms of these genetic factors, integrative analyses using both types of data are important. Even though integration of multiple types of data sets is a promising method as it is generally more powerful than ordinary association studies [3], the method of integration itself is challenging.

Most existing methods use additional information to filter out nonsignificant loci and reduce the total number of tests, which, in return, improve power [4]. On the other hand, mediation analyses usually consider only a single mediator and require multiple testing correction [5]. An example is Zhao et al. [6] who proposed an integrative test, denoted as o-eSNP that was shown to be more powerful than traditional GWAS. Motivated by the data provided by GAW20, in which SNP and DNA methylation data for integrative analysis are available, we aim to characterize the effects of SNPs into direct and indirect effects. As the direct effect of SNPs is simple and straightforward, in this contribution we focus on the indirect effect of SNPs. With the prior knowledge that DNA methylation can be modulated by SNPs [7], we assume that some SNPs exert their effects by regulating the DNA methylation level. Hence the indirect effect of SNPs on a phenotype of interest here is taken as the combined effects of SNPs on DNA methylation and DNA methylation on the phenotype.

In this paper, we propose a novel method, LIPID (likelihood inference proposal for indirect estimation), to use both SNP and DNA methylation data to test whether there is an indirect effect of SNPs on a phenotype. The indirect effect and its variance–covariance matrix are derived, and a Wald test is conducted. An extensive simulation study was done to evaluate the properties and the performance of LIPID, which was also applied to analyze the GAW20 real data.

## Methods

Suppose there are *n* independent subjects, and for each subject, its SNP, DNA methylation, covariates, and phenotype are measured. Specifically, let *Y* = (*Y*_1_,…,*Y*_n_)^*T*^ be the vector of observed phenotypes; *X* = (*X*_1_,…,*X*_k_) be the *n* × *k* matrix denoting the observed values of *k* (nongenetic) covariates, including intercept; *S* = (*S*_1_,..., *S*_r_) and *M* = (*M*_1_,..., *M*_p_) be the *n* × *r* and *n* × *p* matrices regarding the genotypes of *r* SNPs and methylation levels of *p* cytosine-phosphate-guanine (CpG) sites, respectively. Assuming that phenotype *Y* is a continuous variable, we can use a set of linear models to capture the relationship among *Y*, *X*, *S*, and *M* as follows:

1 $$ \mathrm{Y}={\mathrm{S}\upalpha}_{\mathrm{S}}+{\mathrm{M}\upalpha}_{\mathrm{M}}+{\mathrm{X}\upalpha}_{\mathrm{X}}+{\upvarepsilon}_{\mathrm{Y}}, $$

2 $$ \mathrm{M}={\mathrm{S}\upbeta}_{\mathrm{S}}+{\mathrm{X}\upbeta}_{\mathrm{X}}+{\upvarepsilon}_{\mathrm{M}}, $$

where *ε*_*Y*_ *∼ N (0,*$\kern0.3em {\sigma}_Y^2{I}_n$*)*, *vec(ε*_*M*_*) ∼ N (Σ*_*M*_ *⊗I*_*n*_*)*, *vec(·)* is the vectorization operation; *⊗* is the Kronecker product; and *ε*_*Y*_ and *ε*_*M*_ are independent. Note that here *β*_*S*_ and *β*_*X*_ are *r × p* and *k × p* matrices respectively, and Σ_M_ is a *p × p* positive definite matrix. Model (1) characterizes the relationship between phenotype and SNPs, DNA methylation, and covariates, while model (2) depicts the relationship between DNA methylation (as a response variable) and SNPs and covariates. It is concluded from models (1) and (2) that the direct effect of SNPs on the phenotype is *α*_S_ and the indirect effect is *γ* = *β*_S_*α*_M_.

### Estimation and inference

For linear models (1) and (2), we have the following maximum likelihood estimates

$$ \widehat{\alpha}={\left({G}_1^T{G}_1\right)}^{-1}{G}_1^TY,\widehat{\beta}={\left({G}_2^T{G}_2\right)}^{-1}{G}_2^TM $$

where ${G}_1=\left(S,M,X\right),{G}_2=\left(S,X\right),\kern0.5em \alpha ={\left({\alpha}_S^T,{\alpha}_M^T,{\alpha}_X^T\right)}^T,\mathrm{and}\ \beta ={\left({\beta}_S^T,{\beta}_X^T\right)}^T.$ The variance–covariance matrices for $\widehat{\alpha}$and vec $\left(\widehat{\beta}\right)$ are, respectively:

$$ Cov\left(\widehat{\alpha}\right)={\left({G}_1^T{G}_1\right)}^{-1}{\sigma}_Y^2 $$

$$ Cov\left( vec\left(\hat{\beta}\right)\right)={\left({G}_2^T{G}_2\right)}^{-1}\otimes {\Sigma}_M $$

Their corresponding block matrices are the variance–covariance matrices for ${\widehat{\alpha}}_M$ and vec $\left(\widehat{\beta_S}\right)$, respectively. According to the law of total variation, the variance–covariance matrix for $\widehat{\upgamma}$ is

$$ Cov\left(\widehat{\gamma}\right)= Cov\left(\widehat{\beta_S}\widehat{\alpha_M}\right) $$

$$ = Cov\left(E\left({\widehat{\beta}}_S{\widehat{\alpha}}_M|{\widehat{\beta}}_S\right)\right)+E\left( Cov\left({\widehat{\beta}}_S{\widehat{\alpha}}_M|{\widehat{\beta}}_S\right)\right) $$

$$ ={\alpha}_M^T{\varSigma}_M{\alpha}_M{\left({G}_2^T{G}_2\right)}_{11}^{-1}+{\beta}_S Cov\left({\widehat{\alpha}}_M\right){\beta}_S^T+ tr\left({\varSigma}_M Cov\left({\widehat{\alpha}}_M\right)\right){\left({G}_2^T{G}_2\right)}_{11}^{-1} $$

where (·)_11_ represents the first *r* × *r* diagonal submatrix. As *α*_M_ and *β*_S_ are unavailable, their estimates ${\widehat{\alpha}}_M$ and ${\widehat{\beta}}_S$ are used. After several lines of algebra we have

$$ E\left({\widehat{\alpha}}_M^T{\widehat{\varSigma}}_M{\widehat{\alpha}}_M\right)={\alpha}_M^T{\varSigma}_M{\alpha}_M+ tr\left({\varSigma}_M Cov\left({\widehat{\alpha}}_M\right)\right) $$

$$ E\left({\widehat{\beta}}_S Cov\left({\widehat{\alpha}}_M\right){\widehat{\beta}}_S^T\right)={\beta}_S Cov\left({\widehat{\alpha}}_M\right){\beta}_S^T+ tr\left({\varSigma}_M Cov\left({\widehat{\alpha}}_M\right)\right){\left({G}_2^T{G}_2\right)}_{11}^{-1} $$

So an unbiased estimate for the variance–covariance matrix of $\kern0.1em \widehat{\upgamma}$is

$$ \widehat{C} ov\left(\widehat{\gamma}\right)={\widehat{\alpha}}_M^T{\widehat{\varSigma}}_M{\widehat{\alpha}}_M{\left({G}_2^T{G}_2\right)}_{11}^{-1}+{\widehat{\beta}}_S\widehat{C} ov\left({\widehat{\alpha}}_M\right){\widehat{\beta}}_S^T- tr\left({\widehat{\varSigma}}_M\widehat{C} ov\left({\widehat{\alpha}}_M\right)\right){\left({G}_2^T{G}_2\right)}_{11}^{-1}, $$

where ${\widehat{\varSigma}}_M\  and\ \widehat{C} ov\left({\widehat{\alpha}}_M\right)$ are corresponding estimates of Σ_*M*_ and *Cov*$\left({\widehat{\alpha}}_M\right)$. This estimate is different from o-eSNP [6] in the last component. The Wald statistic to test if indirect effect exists is

$$ LIPID={\widehat{\gamma}}^T\widehat{C} ov{\left(\widehat{\gamma}\right)}^{-1}\widehat{\upgamma}, $$

which asymptotically follows a chi-squared distribution with *r* degrees of freedom.

### Adaptation to correlated subjects

For subjects with correlation between each other, linear model with mixed effect is utilized. So model (1) is changed to

$$ \mathrm{Y}={S\alpha}_{\mathrm{S}}+{\mathrm{M}\upalpha}_{\mathrm{M}}+{\mathrm{X}\upalpha}_{\mathrm{X}}+\mathrm{b}+{\upvarepsilon}_{\mathrm{Y}} $$

where *b* is the random effect, with mean 0 and variance–covariance matrix $2{\sigma}_b^2\Phi$, where Φ is the kinship coefficient matrix, and *b* + *ε*_Y_ ∼ $N\left(0,{\sigma}_Y^2{I}_n+2{\sigma}_b^2\varPhi \right).$ Model (2) is not changed. The estimate *α*_*M*_ and its variance–covariance matrix are derived similarly, and the LIPID statistic has the same form.

### Simulation study

To evaluate the performance of LIPID, simulation under various scenarios are conducted. For simplicity, we assume there are no covariates, and that *S*, *M*, and *Y* are all univariate. In addition, the direct effect that we are not interested in does not exist in simulation. The simulated data are generated as follows. First, SNP *S* is generated with a minor allele frequency (MAF) under Hardy-Weinberg equilibrium. Then, DNA methylation *M* is generated from a normal distribution with mean *Sβ*_*S*_ and variance ${\sigma}_M^2$. Finally, phenotypic value *Y* is generated from a normal distribution with mean *Mα*_*M*_ and variance ${\sigma}_Y^2$. The number of individuals is set to be 100 and the number of replications is 10,000. As Table 1 shows, there are 5 scenarios of parameters designed to gauge the Type I error rates of LIPID, and 5 scenarios for the evaluation of power. The variance of *Y* and variance of *M* are fixed to 1 for simplicity. In scenario 1, the coefficients *β*_*S*_ and *α*_*M*_ are both 0; in scenarios 2 and 3, *β*_*S*_ is nonzero while *α*_*M*_ is 0; in scenarios 4 and 5, *α*_*M*_ is nonzero with *β*_*S*_ equal to 0. In these scenarios, the indirect effect is nonexistent, so we change the coefficient of one parameter to measure the Type I error rates under different situations. Under *H*_*a*_, the indirect effect is *β*_*S*_ *α*_*M*_ *≠* 0. The coefficients are chosen so that different methods have moderate powers. From scenario 1 to scenario 5, the *β*_*S*_ and *α*_*M*_ are increased. The MAF ranges from 0.1 to 0.4.

**Table 1:** Parameter settings under H_0_: γ = β_S_α_M_ = 0 and H_a_: γ = β_S_α_M_ ≠ 0

Hypothesis	Parameter	Scenario				
		1	2	3	4	5
*H*_0_	*β*_*S*_	0	0.4	1	0	0
	*α*_*M*_	0	0	0	0.4	1
*H*_a_	*β*_*S*_	0.2	0.3	0.2	0.3	0.4
	*α*_*M*_	0.4	0.4	0.6	0.6	0.6

## Results

**Fig. 1:**
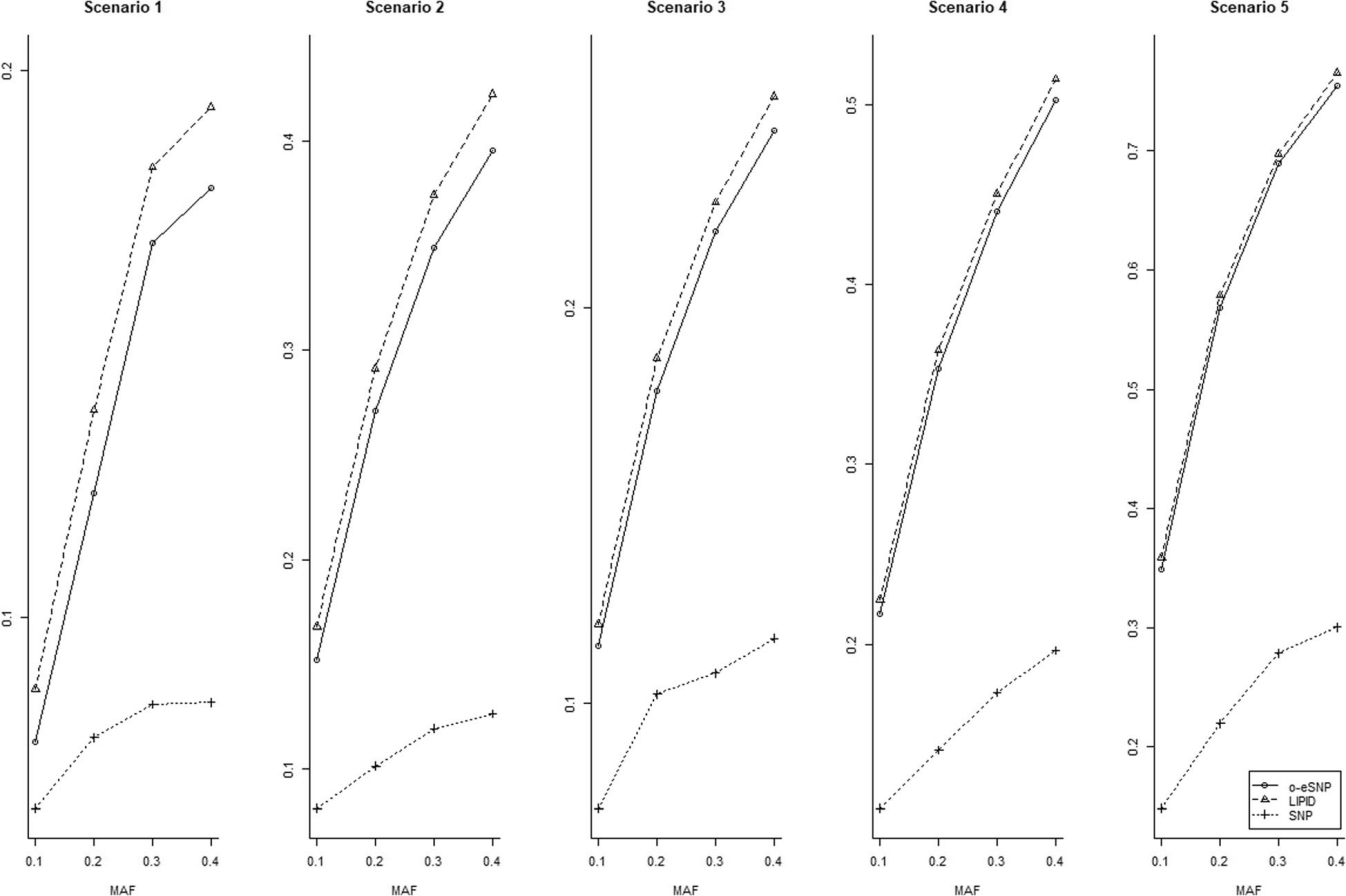
Power for 3 methods under 5 scenarios. The o-eSNP, LIPID, and SNP methods are denoted with ○, Δ, and +, respectively. The MAF ranges from 0.1 to 0.4

Table 2 shows the Type I error rates in the 5 scenarios, from which we can see that the Type I error rates are more or less conservative in scenarios 1 to 5 for o-eSNP and LIPID, while regressing on SNPs only (denoted as SNP in Table 2) controls the Type I error rate well. For scenario 1, where both coefficients *β*_*S*_ and *α*_*M*_ are 0, the Type I error rate is conservative; for scenarios with 1 coefficient that is not 0 but relatively small, the Type I error rates are still conservative; for scenarios with 1 large coefficient, the Type I error rates are better controlled. Compared to o-eSNP [6], the Type I error rates of LIPID are favorable, as o-eSNP is more conservative in all scenarios. Figure 1 shows the powers of 3 methods in 5 scenarios. We can see that LIPID is the most powerful in all scenarios, while SNP-only method has the least power. From scenario 1 to scenario 5, as the indirect effect increases, the performance of LIPID and o-eSNP are very close to each other.

**Table 2:** Type I error rates of 3 methods in scenarios 1 to 5

MAF	Method	Scenario				
		1	2	3	4	5
0.1	o-eSNP	0.000	0.003	0.028	0.025	0.048
	LIPID	0.000	0.004	0.032	0.029	0.049
	SNP	0.054	0.054	0.056	0.051	0.048
0.2	o-eSNP	0.000	0.007	0.039	0.024	0.053
	LIPID	0.000	0.009	0.042	0.028	0.054
	SNP	0.056	0.053	0.053	0.056	0.054
0.3	o-eSNP	0.000	0.009	0.041	0.027	0.050
	LIPID	0.000	0.011	0.044	0.032	0.051
	SNP	0.059	0.054	0.052	0.053	0.057
0.4	o-eSNP	0.000	0.011	0.043	0.021	0.048
	LIPID	0.000	0.013	0.044	0.025	0.049
	SNP	0.052	0.056	0.056	0.051	0.054

The MAF changes from 0.1 to 0.4

### Real data analysis

The GAW20 real data package contains genomic, DNA methylation, and phenotypic data for more than 1000 individuals from 188 pedigrees. The phenotypic data include metabolic indices, lipoproteins, and triglyceride. Dense genome-wide SNP markers make up the genomic data. DNA methylation levels are also available on CpG sites genome-wide before and after individuals are treated with fenofibrate. The level of triglycerides and the methylation level before treatment are made use of. The covariates include gender, age, smoking status, high-density lipoprotein, metabolic disorder, and center. We use SOLAR (Sequential Oligogenic Linkage Analysis Routines) [8] to obtain the heritability for triglyceride level, using 1108 subjects with phenotypic data. The total number of subjects with SNP data and DNA methylation is 716. As LIPID considers a region of multiple genetic markers, SNPs and DNA methylation loci within the range of each gene are analyzed; we analyze a total of 13,968 genes. Genes that pass false discovery rate (FDR) correction are *FAT1* (*p* value 9.4E-7) and *DCTN6* (*p* value 1.3E-6), while o-eSNP fails to find any significant genes (*FAT1 p* value 2.5E-5; *DCTN6 p* value 3.7E-6). We further use the BIOS QTL (quantitative trait locus) browser [9] to validate our findings. We found that rs458021 on gene *FAT1* is a *cis*-meQTL (methylation quantitative trait locus) with a *p* value of 3.8E-07, but we did not find any meQTL on gene *DCTN6*. *FAT1* is associated with cholesterol in DAVID (Database for Annotation, Visualization, and Integrated Discovery), whereas *DCTN6* is involved in lipid metabolism [10]. Because the eQTM (expression quantitative trait methylation) database are not widely available, we cannot further validate if these CpG sites on these genes can modulate the expression, and further influence the phenotype.

## Discussion

Compared to o-eSNP [6], LIPID controls Type I error rates better in all scenarios, and the power is higher in all scenarios. The estimate itself is the same, no matter if we regress *M* or *Mα*_*M*_ on *S*, but the variance–covariance estimates are different, and LIPID has a less-biased variance–covariance estimate, which leads to the improvement of performance of LIPID. Furthermore, we also adapt o-eSNP and LIPID to correlated subjects.

Application of LIPID to the GAW20 real data indicates that LIPID is capable of detecting genes with indirect effect. The computation is efficient, and the process takes 30 min to analyze the whole GAW20 data set on a personal computer with an Intel Core i3–4150 CPU. The genes identified appear to be functionally relevant to the trait being considered, thereby substantiating the importance of these findings and leading to confidence of genes found being true discoveries.

## Conclusions

For complex diseases, we propose a novel method to detect indirect effect of SNPs on a phenotype via methylation, and we demonstrate its superiority compared to 2 existing methods. LIPID is single-step and does not require multiple tests, compared to traditional mediation analysis; at the same time, multiple genetic loci can be used simultaneously to test indirect effect.

## Abbreviations

DAVID: Database for annotation, visualization, and integrated discovery
eQTM: Expression quantitative trait methylation
FDR: False discovery rate
GAW: Genetic analysis workshop
LIPID: Likelihood inference proposal for indirect estimation
MAF: Minor allele frequency
meQTL: Methylation quantitative trait locus
QTL: Quantitative trait locus
SOLAR: Sequential oligogenic linkage analysis routines
